# Assessment of colon cancer molecular mechanism: a system biology approach 

**Published:** 2021

**Authors:** Babak Arjmand, Mahmood Khodadoost, Somayeh Jahani Sherafat, Mostafa Rezaei Tavirani, Nayebali Ahmadi, Maryam Hamzeloo Moghadam, Sina Rezaei Tavirani, Binazir Khanabadi, Majid Iranshahi

**Affiliations:** 1 *Cell Therapy and Regenerative Medicine Research Center, Endocrinology and Metabolism Molecular-Cellular Sciences Institute, Tehran University of Medical Sciences, Tehran, Iran*; 2 *School of Traditional Medicine Shahid, Beheshti University of Medical Sciences, Tehran, Iran*; 3 *Laser Application in Medical Sciences Research Center, Shahid Beheshti University of Medical Sciences, Tehran, Iran*; 4 *Proteomics Research Center, Faculty of Paramedical Sciences, Shahid Beheshti University of Medical Sciences, Tehran, Iran*; 5 *Traditional Medicine and Materia Medica Research Center, School of Traditional Medicine, Shahid Beheshti University of Medical Sciences, Tehran, Iran*; 6 *Proteomics Research Center, Shahid Beheshti University of Medical Sciences, Tehran, Iran*; 7 *Gastroenterology and Liver Diseases Research Center, Research Institute for Gastroenterology and Liver Diseases, Shahid Beheshti University of Medical Sciences, Tehran, Iran*

**Keywords:** Colon cancer, Protein expression, Bioinformatics, Human, Network analysis

## Abstract

**Aim:**

The current study aimed to assess and compare colon cancer dysregulated genes from the GEO and STRING databases.

**Background:**

Colorectal cancer is known as the third most common kind of cancer and the second most important reason for global cancer-related mortality rates. There have been many studies on the molecular mechanism of colon cancer

**Methods:**

From the STRING database, 100 differentially expressed proteins related to colon cancers were retrieved and analyzed by network analysis. The central nodes of the network were assessed by gene ontology. The findings were compared with a GSE from GEO.

**Results:**

Based on data from the STRING database, TP53, EGFR, HRAS, MYC, AKT1, GAPDH, KRAS, ERBB2, PTEN, and VEGFA were identified as central genes. The central nodes were not included in the significant DEGs of the analyzed GSE.

**Conclusion:**

A combination of different database sources in system biology investigations provides useful information about the studied diseases.

## Introduction

 Colorectal cancer is known as the third most common kind of cancer and the second most important reason for global cancer-related mortality rates (1). It is one of the lethal cancers that is associated with problems in diagnosis as well as therapy (2). Many investigations into colon cancer and its molecular mechanism have been performed using different methods (3). Because high throughput methods are widely common in different fields of medical sciences, there are many documents about colon cancer which are concerned with high throughput methods (4). Proteomics, genomics, and bioinformatics are the important high throughput methods that are tied together to solve various problems in medical research. The results of several investigations into colon cancer that were administrated by proteomics, genomics, metabolomics, or bioinformatics have been published. Bioinformatics is a critical field applied to create new concepts by using the analysis results of genomic and proteomic studies (5-7). 

Dysregulated metabolites, genes, and proteins in colon cancer patients have been studied using bioinformatics. In such studies, much is gathered from databanks or published articles and analyzed using bioinformatic tools (8-10). First, the diversity of data sources, and second, the multiplicity of analysis methods are interesting points about these studies. Based on the selected source and method of investigation, results can be different. It seems clear that an explanation of the investigation protocol is required to determine the most accurate findings (8, 11).

GEO is a useful source of data, including gene expression profiles of assessed samples. Many researchers select GEO as a source of data to analyze differentially expressed genes in a defined condition. GEO is not only suitable source of data, but it is also equipped with useful software such as GEO2R which helps the primary analysis of data. Fold change and statistical validation of data are two important findings from GEO. The style of gene regulation, i.e. up- or downregulation is accessible in GEO2R analysis of the studied DEGs (12, 13).

STRING is another useful source of data that provides the related dysregulated proteins in the studied condition. There are many published articles that are concerned with “disease query” of string. Combination of STRING and Cytoscape software is a powerful tool in the bioinformatic analysis of data (14, 15). In the present investigation, dysregulated genes in human colon cancer were assessed by using one recorded experiment in GEO and STRING sources to elucidate the findings. 

## Methods

In this study, 100 proteins associated with colon cancer were extracted from the STRING database using the “disease query option.” The proteins were interacted by Cytoscape software v 3.7.2 (16) by undirected edges, and the network comprising 100 nodes and 2811 links was constructed. The main connected components, including 95 nodes and 5 isolated proteins, were analyzed by the “NetworkAnalyzer” plug in of Cytoscape software. The network was visualized based on degree value by considering the color and size of the nodes.

Based on degree value, the 10 top nodes of the main connected component were selected as the hub nodes of the network. The hubs were included in the ClueGO v2.5.7 (17) application of Cytoscape to analyze gene ontology. The related pathways were extracted from KEGG 08.05.2020. A *p-*value ≤ 0.01 and network specificity; medium were applied to determine the pathways.

The GSE127069 of 6 patients, entitled “RNA sequencing for cancer tissues and adjacent tissues of third-stage rectal cancer patients with and without blood vascular thrombus” in GEO (18) was selected for analysis. The volcano plot of gene expression profiles of colon cancer tissue versus adjacent tissue was provided to statistically match the data. The top genes based on fold change (1.5<FC<-1.5) and *p*-value < 0.01 were selected as significant DEGs. The known genes were identified based on gene IDs from Uniport (https://www.uniprot.org). 

## Results

**Figure 1:**
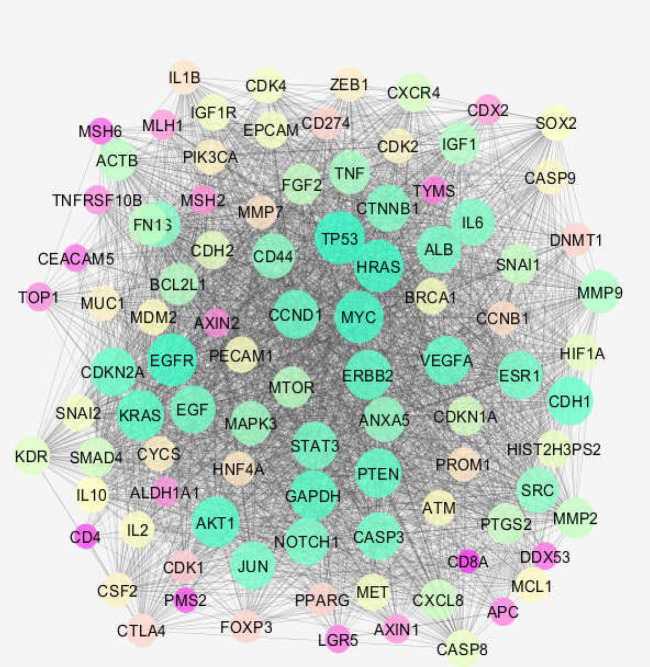
Main connected component of colon cancer network. Among 100 extracted proteins from STRING database, 95 individuals are included in the subnetwork. The nodes are layout base on degree value; bigger size and red to green refer to increment of degree value

The network, including a main connected component (shown in Figure 1) and 5 isolated proteins, was constructed for the extracted data from the STRING database. Four centrality parameters, i.e. degree (K), betweenness centrality (BC), closeness centrality (CC), and stress, were determined for the nodes of the main connected component (Table 1). TP53, EGFR, HRAS, MYC, AKT1, GAPDH, KRAS, ERBB2, PTEN, and VEGFA were identified as hub nodes. Thirty-one dysregulated terms in 2 groups of pathways which were related to the hub nodes of the colon cancer network were identified. The pathways that are classified in the two groups and the related proteins are presented in Table 2. 

**Figure 2:**
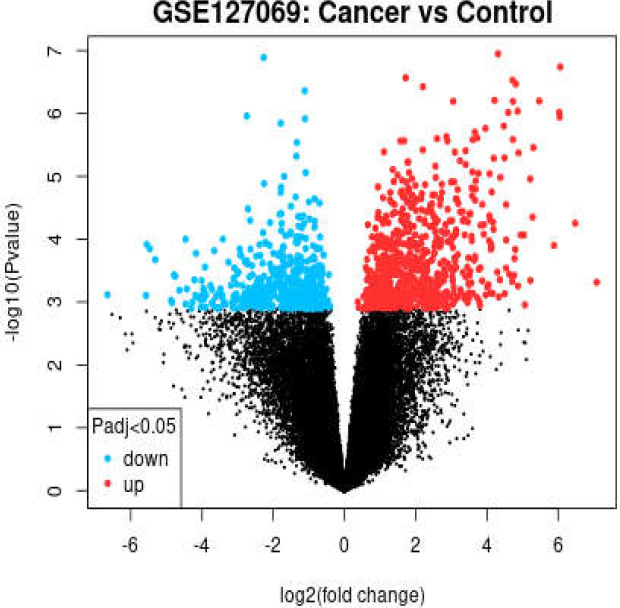
Volcano plot of gene expression profiles of colon cancer tissue versus adjacent tissue

The volcano plot of gene expression profiles of colon cancer tissue versus adjacent tissue for the analyzed GSE is presented in Figure 2. Based on the volcano plot, the samples are comparable. A list of the significant and known genes of the GEO analysis is given in Table 3. The top 21 rows of Table 3 refer to the downregulated genes, and the other 6 genes are upregulated.

**Table 1:** The nodes of the main connected component and four centrality parameters are presented

R	display name	Degree	Betweenness Centrality	Closeness Centrality	Stress
1	TP53	92	0.016	0.979	3026
2	EGFR	91	0.018	0.969	3038
3	HRAS	91	0.015	0.969	2940
4	MYC	90	0.014	0.959	2758
5	AKT1	87	0.011	0.931	2410
6	GAPDH	86	0.010	0.922	2248
7	KRAS	86	0.012	0.922	2444
8	ERBB2	85	0.011	0.913	2268
9	PTEN	84	0.011	0.904	2178
10	VEGFA	84	0.009	0.904	2020
11	CCND1	83	0.009	0.895	2026
12	CDH1	83	0.008	0.895	1982
13	CDKN2A	83	0.010	0.895	2034
14	CASP3	81	0.007	0.879	1748
15	EGF	81	0.009	0.879	1840
16	ESR1	81	0.008	0.879	1806
17	JUN	81	0.011	0.879	1952
18	STAT3	81	0.006	0.879	1638
19	ALB	80	0.006	0.870	1582
20	NOTCH1	80	0.007	0.870	1772
21	CTNNB1	79	0.009	0.862	1802
22	IL6	79	0.008	0.862	1628
23	INS	77	0.004	0.847	1290
24	CD44	76	0.009	0.839	1548
25	SRC	76	0.006	0.839	1368
26	ANXA5	75	0.004	0.832	1228
27	MAPK3	73	0.003	0.817	1030
28	TNF	72	0.007	0.810	1214
29	MMP9	71	0.003	0.803	898
30	IGF1	70	0.003	0.797	848
31	BCL2L1	69	0.003	0.790	846
32	ACTB	68	0.003	0.783	830
33	MTOR	68	0.002	0.783	748
34	FGF2	67	0.002	0.777	738
35	FN1	67	0.002	0.777	702
36	MMP2	67	0.002	0.777	718
37	PTGS2	66	0.003	0.770	808
38	SNAI1	66	0.003	0.770	792
39	CXCL8	65	0.002	0.764	624
40	CDKN1A	63	0.003	0.752	668
41	CXCR4	63	0.003	0.752	598
42	SMAD4	63	0.005	0.752	912
43	KDR	62	0.002	0.746	556
44	CDH2	61	0.002	0.740	646
45	CASP8	60	0.002	0.734	508
46	HIF1A	60	0.001	0.734	406
47	HIST2H3PS2	60	0.002	0.734	604
48	CDK4	58	0.002	0.723	576
49	EPCAM	58	0.005	0.723	854
50	IGF1R	58	0.001	0.723	362
51	MET	58	0.002	0.723	458
52	SNAI2	58	0.002	0.723	448
53	BRCA1	57	0.004	0.718	694
54	IL2	57	0.004	0.718	662
55	PECAM1	57	0.001	0.718	390
56	SOX2	57	0.002	0.718	542
57	ATM	56	0.003	0.712	556
58	IL10	56	0.003	0.712	530
59	MDM2	56	0.001	0.712	418
60	MCL1	55	0.001	0.707	314
61	CASP9	54	0.001	0.701	332
62	CDK2	54	0.003	0.701	562
63	CSF2	53	0.003	0.696	534
64	CYCS	53	0.002	0.696	386
65	MUC1	53	0.002	0.696	516
66	PIK3CA	53	0.007	0.696	830
67	ZEB1	52	0.001	0.691	286
68	HNF4A	51	0.002	0.686	370
69	PROM1	51	0.001	0.686	216
70	IL1B	50	0.002	0.681	354
71	MMP7	50	0.001	0.681	296
72	CCNB1	49	0.001	0.676	300
73	CTLA4	47	0.002	0.667	406
74	CD274	46	0.003	0.662	484
75	DNMT1	46	0.002	0.662	418
76	FOXP3	46	0.003	0.662	420
77	PPARG	46	0.001	0.662	180
78	CDK1	44	0.001	0.653	208
79	CDX2	34	0.001	0.606	238
80	MLH1	34	0.002	0.610	272
81	ALDH1A1	32	0.000	0.603	56
82	AXIN1	32	0.000	0.603	74
83	MSH2	32	0.001	0.603	216
84	TNFRSF10B	31	0.000	0.599	30
85	AXIN2	30	0.000	0.595	88
86	TOP1	30	0.001	0.595	112
87	APC	28	0.001	0.588	124
88	LGR5	26	0.000	0.580	76
89	TYMS	26	0.001	0.577	116
90	CEACAM5	25	0.001	0.577	130
91	DDX53	24	0.000	0.573	16
92	MSH6	22	0.000	0.566	62
93	PMS2	17	0.000	0.550	10
94	CD4	16	0.000	0.547	38
95	CD8A	11	0.000	0.525	14

## Discussion

**Table 2:** The biochemical pathways which are related to the 10 hub nodes are presented. Term p-value, Term p-value Corrected with Bonferroni step down, group p-value, and group p-value Corrected with Bonferroni step down were 0.00. %AG, Nr. G, and AG refer to percentage of associated genes, number of associated genes, and associated genes respectively. Highlighted row refers to the first group and the other terms belong to the second group (Endometrial group)

GOTerm	% AG	Nr. G	AG
HIF-1 signaling pathway	5	5	[AKT1, EGFR, ERBB2, GAPDH, VEGFA]
ErbB signaling pathway	7	6	[AKT1, EGFR, ERBB2, HRAS, KRAS, MYC]
Sphingolipid signaling pathway	4	5	[AKT1, HRAS, KRAS, PTEN, TP53]
Mitophagy	4	3	[HRAS, KRAS, TP53]
Longevity regulating pathway	4	4	[AKT1, HRAS, KRAS, TP53]
Longevity regulating pathway	5	3	[AKT1, HRAS, KRAS]
VEGF signaling pathway	7	4	[AKT1, HRAS, KRAS, VEGFA]
Fc epsilon RI signaling pathway	4	3	[AKT1, HRAS, KRAS]
Prolactin signaling pathway	4	3	[AKT1, HRAS, KRAS]
Thyroid hormone signaling pathway	4	5	[AKT1, HRAS, KRAS, MYC, TP53]
GnRH secretion	5	3	[AKT1, HRAS, KRAS]
AGE-RAGE signaling pathway in diabetic complications	4	4	[AKT1, HRAS, KRAS, VEGFA]
Colorectal cancer	7	6	[AKT1, EGFR, HRAS, KRAS, MYC, TP53]
Renal cell carcinoma	6	4	[AKT1, HRAS, KRAS, VEGFA]
Pancreatic cancer	8	6	[AKT1, EGFR, ERBB2, KRAS, TP53, VEGFA]
Endometrial cancer	14	8	[AKT1, EGFR, ERBB2, HRAS, KRAS, MYC, PTEN, TP53]
Glioma	8	6	[AKT1, EGFR, HRAS, KRAS, PTEN, TP53]
Prostate cancer	7	7	[AKT1, EGFR, ERBB2, HRAS, KRAS, PTEN, TP53]
Thyroid cancer	11	4	[HRAS, KRAS, MYC, TP53]
Melanoma	8	6	[AKT1, EGFR, HRAS, KRAS, PTEN, TP53]
Bladder cancer	17	7	[EGFR, ERBB2, HRAS, KRAS, MYC, TP53, VEGFA]
Chronic myeloid leukemia	7	5	[AKT1, HRAS, KRAS, MYC, TP53]
Acute myeloid leukemia	6	4	[AKT1, HRAS, KRAS, MYC]
Small cell lung cancer	4	4	[AKT1, MYC, PTEN, TP53]
Non-small cell lung cancer	9	6	[AKT1, EGFR, ERBB2, HRAS, KRAS, TP53]
Breast cancer	5	8	[AKT1, EGFR, ERBB2, HRAS, KRAS, MYC, PTEN, TP53]
Hepatocellular carcinoma	4	7	[AKT1, EGFR, HRAS, KRAS, MYC, PTEN, TP53]
Gastric cancer	5	7	[AKT1, EGFR, ERBB2, HRAS, KRAS, MYC, TP53]
Central carbon metabolism in cancer	12	8	[AKT1, EGFR, ERBB2, HRAS, KRAS, MYC, PTEN, TP53]
Choline metabolism in cancer	4	4	[AKT1, EGFR, HRAS, KRAS]
PD-L1 expression and PD-1 checkpoint pathway in cancer	6	5	[AKT1, EGFR, HRAS, KRAS, PTEN]

Many diseases contained in the STRING database have related dysregulated proteins listed. In this research, 100 proteins that are dysregulated in human colon cancers were retrieved. The data was organized in the protein-protein interaction unit (Figure 1). The constructed network analysis revealed that the network is a scale-free network, in which the number of limited nodes which are known as central nodes can be selected as critical nodes of the analyzed network (19). As shown in Table 1, the centrality parameters of nodes were determined. TP53, EGFR, HRAS, MYC, AKT1, GAPDH, KRAS, ERBB2, PTEN, and VEGFA are appeared as hub nodes of the assessed network. The hub genes are the important central nodes that can be discriminated from the other nodes of the network as critical individuals (20).

 As shown in Table 1, the other centrality parameters of the hub nodes are also high values; thus, it can be concluded that the hub nodes are potent hub-bottleneck nodes. A usual and simple analysis of data was conducted to find the critical nodes of the studied network. As represented in Table 2, the related pathways for the central nodes were identified through gene ontology analysis. It seems that a complete analysis of data is formed, and a useful interpretation is accessible. 

Based on previous investigations, TP53 is the top central gene related to colon cancer and known as a biomarker of many cancers (21). As specificity and sensitivity are the two main properties of biomarkers (22), it can be concluded that TP53 cannot be considered as a biomarker of colon cancer. Like TP53, the other introduced critical nodes are also related to different types of cancers. Thus, it can be concluded that the well-known data in the STRING database can be matched with various kinds of cancers. As reported, EGFR is a key element in colorectal cancers (23), and many documents point to EGFR as a biomarker of cancers such as head and neck squamous cell carcinomas and primary non-small cell lung cancer (24, 25). In another part of the study, colon cancer tissue was compared with adjacent tissues. As depicted in Figure 2, the data indicated that analysis is possible. In total, 27 significant DEGs that discriminate cancerous tissue from the adjacent tissue were identified. In the first attempt, it was concluded that the evidence for a correlation between the findings and the results of STRING analysis is insufficient (Compare the contents of Table 3 and the introduced 10 central nodes). As the number of DEGs in the GEO analysis is limited to 27, inclusion of data in an interactome cannot be conducted to form a scale-free network. 

**Table 3:** Significant and known genes of the GEO analysis are presented. Among the 27 genes the top 21 DEGs are down-regulated and the other 6 genes are upregulated

R	Spot ID	Gene name
1	P52761	slr0709
2	P57784	Snrpa1
3	P49155	xylI
4	P57417	flgN
5	P56746	CLDN15
6	P80162	CXCL6
7	P47148	PXP2
8	P96036	spt5
9	P20443	Sag
10	P24716	copR
11	P60079	MW2494
12	P44648	trmB
13	P62115	psbN
14	P94795	nifH
15	P26439	HSD3B2
16	P88119	env
17	P96403	MT0231
18	P63777	citA
19	P75978	ymfN
20	P62741	HBG1
21	P56001	rpoA
22	P41292	MT-ATP8
23	P67259	NMB0796
24	P59988	uspB
25	P82762	LCR47
26	P52119	ratB
27	P68097	CYCS

The best way to analyze this set of genes is to add their first neighbors. STRING is a rich source of neighbors, and there are options in STRING that allow researchers to add an adequate number of the first neighbors to the queried genes. This mode of analysis enables the investigator to construct a scale-free network and analyze the queried DEGs. The discriminated values of centrality parameters for the queried genes, which were induced by the added first neighbors in addition to the fold change values, provide a clear concept for selecting the critical DEGs from among the studied genes. It can be concluded that each type of analysis is unique in its properties and findings. Based on researcher favorites, a study can be designed to obtain a different result that is useful from that point of view. 

Many studies have been concerned with this combination mode of analysis with different numbers of added first neighbors to discriminate the queried DEGs (26, 27).

The analysis of data from GEO and STRING sources revealed that each kind of analysis has its benefits; however, analysis using the sources separately also provided useful results. It seems that the combination mode of analysis is a suitable and more complete method for finding a clear concept and interpretation of the studied disease.

## Conflict of interests

The authors declare that they have no conflict of interest.
